# Class I HDAC inhibition reduces DNA damage repair capacity of *MYC*-amplified medulloblastoma cells

**DOI:** 10.1007/s11060-023-04445-w

**Published:** 2023-10-03

**Authors:** Johanna Vollmer, Jonas Ecker, Thomas Hielscher, Gintvile Valinciute, Johannes Ridinger, Nora Jamaladdin, Heike Peterziel, Cornelis M. van Tilburg, Ina Oehme, Olaf Witt, Till Milde

**Affiliations:** 1 Hopp Children’s Cancer Center (KiTZ), Im Neuenheimer Feld 430, 69120 Heidelberg, Germany; 2 National Center for Tumor Diseases (NCT), NCT Heidelberg, a partnership between DKFZ and Heidelberg University Hospital, Im Neuenheimer Feld 460, 69120 Heidelberg, Germany; 3 Clinical Cooperation Unit Pediatric Oncology, German Cancer Research Center (DKFZ) and German Consortium for Translational Cancer Research (DKTK), Im Neuenheimer Feld 280, 69120 Heidelberg, Germany; 4 Department of Pediatric Hematology and Oncology, Heidelberg University Hospital, Im Neuenheimer Feld 430, 69120 Heidelberg, Germany; 5 Division of Biostatistics, German Cancer Research Center (DKFZ), and German Cancer Consortium (DKTK), Im Neuenheimer Feld 280, 69120 Heidelberg, Germany; 6 Present Address: Department of Tumor Cell Biology, St Jude Children’s Research Hospital, Memphis, TN USA

**Keywords:** Medulloblastoma, MYC, DNA-damage, HDAC, Synergy

## Abstract

**Purpose:**

*MYC*-driven Group 3 medulloblastoma (MB) (subtype II) is a highly aggressive childhood brain tumor. Sensitivity of *MYC*-driven MB to class I histone deacetylase inhibitors (HDACi) has been previously demonstrated in vitro and in vivo. In this study we characterize the transcriptional effects of class I HDACi in *MYC*-driven MB and explore beneficial drug combinations.

**Methods:**

*MYC*-amplified Group 3 MB cells (HD-MB03) were treated with class I HDACi entinostat. Changes in the gene expression profile were quantified on a microarray. Bioinformatic assessment led to the identification of pathways affected by entinostat treatment. Five drugs interfering with these pathways (olaparib, idasanutlin, ribociclib, selinexor, vinblastine) were tested for synergy with entinostat in WST-8 metabolic activity assays in a 5 × 5 combination matrix design. Synergy was validated in cell count and flow cytometry experiments. The effect of entinostat and olaparib on DNA damage was evaluated by γH2A.X quantification in immunoblotting, fluorescence microscopy and flow cytometry.

**Results:**

Entinostat treatment changed the expression of genes involved in 22 pathways, including downregulation of DNA damage response. The PARP1 inhibitors olaparib and pamiparib showed synergy with entinostat selectively in *MYC*-amplified MB cells, leading to increased cell death, decreased viability and increased formation of double strand breaks, as well as increased sensitivity to additional induction of DNA damage by doxorubicin. Non-*MYC*-amplified MB cells and normal human fibroblasts were not susceptible to this triple treatment.

**Conclusion:**

Our study identifies the combination of entinostat with olaparib as a new potential therapeutic approach for *MYC*-driven Group 3 MB.

**Supplementary Information:**

The online version contains supplementary material available at 10.1007/s11060-023-04445-w.

## Introduction

Medulloblastoma (MB) is one of the most frequent malignant brain tumors in children, accounting for around 10% of all childhood brain tumors [1]. The prognosis of MB patients is very much dependent on the molecular subgroup of the tumor (WNT, SHH, Group 3 and Group 4) [2, 3]. Group 3 and Group 4 tumors show a profound molecular heterogeneity and can be classified into 8 subtypes [4]. Patients with Group 3 MB tumors with high expression of the oncogenic transcription factor MYC (subtype II) [4] show poor survival rates despite highly aggressive treatment. New treatment approaches for this high-risk subset of patients are urgently needed [5].

We and others have previously shown that group 3 MBs have an aberrantly high expression of class I histone deacetylase 2 (HDAC2) [6, 7] and that *MYC*-amplified group 3 MB cells are highly sensitive to class I HDAC inhibition in vitro and in vivo [6, 8]. The cytotoxic effect of class I HDACi in *MYC-*amplified MB is partially mediated by inhibition of HDAC2 in the MYC-HDAC2-protein complex leading to reduced MYC protein turnover and to an alteration of MYC chromatin binding patterns inducing an altered expression of MYC regulated genes [9].

HDACis are established drugs for the treatment of hematological malignancies, currently four HDACi are FDA-approved for the treatment of T-cell lymphoma and multiple myeloma [10]. However, in solid tumors single agent HDACi fail to achieve significant anti-tumor effects in clinical trials [11]. It has become clear that a lack of knowledge on the molecular downstream effect of HDAC inhibition and lack of biomarkers for patient selection are currently impeding the translation of promising preclinical findings into clinical application [12]. It may be that combination therapies will increase the clinical efficacy of HDACis in solid tumors [13].

In this study, we investigate alterations of gene expression in response to class I HDAC inhibition in *MYC*-amplified MB cells to identify affected pathways. Clinically advanced compounds that target these pathways are evaluated for synergistic interaction with entinostat.

## Materials and methods

### Pediatric MB cell lines and culture conditions

HD-MB03 is a cell line established in our laboratory [14]. The MB cell lines ONS76: CVCL_1624, UW228-2: CVCL_0572, MED8A: CVCL_M137, D425: CVCL_1275 [6], as well as the non-transformed human foreskin fibroblast cell line VH7 [15] were obtained and cultured as previously mentioned. All cell lines were authenticated using Multiplex Cell Line Authentication (MCA) and tested for contamination by Multiplex cell Contamination Testing (McCT) by Multiplexion (Heidelberg, Germany) as described before [9]. Cultured cells were checked weekly with PlasmoTest™- (InvivoGen) and monthly with Venor® GenM Classic—(Minerva Biolabs) Mycoplasma Detection Kits for mycoplasma contamination. *MYC* amplification status of cell lines were verified previously [6].

### Gene expression microarray, GSEA, pathway analysis

HD-MB03 were treated in three biological replicates for 24 h and 48 h with 1 µM entinostat or DMSO solvent control. RNA extraction was done with RNeasy Mini Kit (Qiagen) according to the manufacturer’s instructions. RNA quantity was assessed with Nanodrop ND-1000. Microarray-based gene expression profile analysis was performed at the Genomics and Proteomics Core Facility at the German Cancer Research Center (DKFZ) including sample preparation (quality control, reverse transcription, labelling and fragmentation) and data preprocessing. Sample hybridization was done on Human Genome U133 Plus 2.0 Affymetrix microarray according to the manufacturer’s instruction. After preprocessing Affymetrix CEL files were GCRMA normalized and log_2_-transformed. Differentially expressed probesets (adj. p-value < 0.05) between treatments were identified using the empirical Bayes approach based on moderated t-statistics as implemented in the Bioconductor package limma. For comparisons based on multiple time points, a time point specific effect was accounted for in the linear model by performing a two-way ANOVA. Probesets showing an over-additive effect were identified by testing the interaction between time point and treatment by preforming a two-way ANOVA with cross-effects. Gene set enrichment analysis for KEGG, reactome and GO databases was performed using the camera test. In case a gene was represented by multiple probesets, the probeset with the strongest effect was selected for Gene Set Enrichment Analysis (GSEA). P-values were adjusted for multiple testing using the Benjamini–Hochberg correction in order to control the false discovery rate. All analyses were performed with statistical software R 3.6 [16]. Visualization of functional enrichment in networks was carried out using the Cytoscape plug-in EnrichmentMap [17]. Enriched genesets of the three databases with FDR < 0.1 served as input. Assembled networks and clusters were manually re-organized for biofunctional annotation. Additionally differentially expressed genes were analyzed through the use of Ingenuity Pathway Analysis (IPA) to identify enriched canonical pathways of the IPA database (IPA core-analysis) (QIAGEN Inc., https://www.qiagenbioinformatics.com/products/ingenuity-pathway-analysis) [18]. Results from Cytoscape EnrichmentMap and IPA were summarized by manual grouping into overarching pathways representing circumscribed biological functions (Suppl. Table 4).

### Drugs

The following compounds were used: entinostat (Biomol GmbH, M4693-15A.25), idasanutlin (TargetMol; T6254), olaparib (BIOZOL; S1060), ribociclib (supplier), selinexor (BioCat; T6106-TM), vinblastinesulfate (Selleckchem; S4505), doxorubicin in 0.9% NaCl (UKHD pharmacy), niraparib (Selleckchem; S2741), pamiparib (Selleckchem; S8592), veliparib (Selleckchem; S1004). The translational drug library for initial drug selection comprised 74 compounds [19].

### Metabolic activity assay

Cells were seeded in 96-well plates for dose–response investigation and matrix synergism analysis as previously reported [20], drugs were added with the D300e Digital Dispenser (Tecan). Metabolic activity was assessed after 72 h of treatment using the WST-8 based Cell counting Kit-8 (Dojindo, CK04-13) as previously reported [6].

### Synergism-antagonism matrix-treatments and computation

72 h combination treatments of entinostat with idasanutlin, olaparib, ribociclib, selinexor and vinblastine on 96-well cell culture plates included 38 treatment conditions per combination in form of a 5 × 5 checkerboard matrix, applying DMSO normalization and plate randomization [21]. The 5 × 5 concentration matrix was built applying twofold dilution series surrounding each drug’s EC50 as previously reported [21]. After 72 h of treatment metabolic activity was assessed by WST-8 assay. Entinostat dose–response-curve (DRC) shift was analyzed in the presence of a fixed dose of the investigated combination drug. Fixed dose level was set to a concentration between single drug IC20-IC50. DRC left-shift was assessed visually and assigned to increased entinostat potency (i.e. comparable effects were achieved at lower concentrations). CompuSyn software (ComboSyn Inc., Paramus, NJ, USA) was used to calculate combination index (CI) values at three constant ratio combinations including an equipotent ratio-combination (EC 50: EC 50). SynergyFinder 1.0 web application was used to calculate synergism across the whole dose–response surface applying the ZIP synergy model [21].

### Cell counts and viability assessments

Cell counts and viability were assessed using Vi-CELL™ Cell Viability Analyzer (Beckman Coulter) as previously described [6]. Treatment with 500 nM staurosporine served as control for maximal achievable effects. Combination index (CI) values were calculated using CompuSyn-Software (ComboSyn Inc., Paramus, NJ, USA).

### Protein isolation and western blot analysis

Western blots were performed as previously described [9]. The following antibodies were used: c-MYC (Abcam; ab32072; 1:5000), PARP1 (CellSignalling; #9532S; 1:5000), Rad51 (GeneTex; GTX70230; 1:5000), γH2A.X (CellSignalling; #9718; 1:2000), beta-actin (Sigma; A5441; 1:10,000). Immunodetection was done by luminescence using Amersham ECL Prime Western Blotting Detection Reagent (GE Healthcare) with Azure Azure 400 imaging system (Azure biosystems).

### Cell cycle analysis and flow cytometric analysis of DNA double strand breaks

Equal amounts of cells were fixed by dropwise addition of ice-cold 70% ethanol and incubated for 2 h at 4 °C. For cell cycle analysis cells were washed in 1% BSA – PBS – 0.2% Tween20 and subsequently incubated in PI staining solution (50 µg/ml PI, 5 µg/ml RNAse in 1% BSA—PBS—0.2% Tween20) for 20 min at 37 °C under light protection. Assessment of γH2A.X was done as previously described [22]. Flow cytometric analysis was done on FACS Canto II. Data analysis was performed using FlowJo software. After doublet exclusion using a SSC-A/SSC-W plot, cell cycle phases were assigned. γH2A.X high gates were set to contain ~ 5% of cells in the untreated condition representing intrinsic DNA damage. Mean normalized fluorescence intensity was calculated and normalized as previously reported [22].

### Immunofluorescence microscopy of γH2A.X staining

Cells were seeded at a density of 30,000 per chamber into a µ-Slide 8 Well (Ibidi) 24 h before treatment. After 24 h treatment cells were washed with PBS and fixation was performed with 4% PFA for 20 min at room temperature. Fixed cells were washed with PBS, permeabilized with 0.2% Triton-X-100 in PBS for 30 min and blocked with 0.05% Triton-X-100—3% BSA—PBS for 1 h at room temperature. After incubation with γH2A.X antibody (1:200) at 4 °C overnight cells were incubated with Alexa-Fluor-488-labeled secondary antibody (ThermoFisher; A-11008; 1:400) for 2 h at room temperature and counterstained with DAPI (4′,6-Diamidin-2-phenylindol) for 10 min. Images were acquired on a Zeiss LSM 710 laser scanning confocal microscope (Oberkochen, Germany) with 40 × times magnification. Exemplary γH2A.X foci quantification was done semi-automatically using ImageJ for > 100 nuclei per condition for two biological duplicates.

### Gene expression and protein expression data of primary medulloblastoma samples, statistical analysis and graph editing

Primary mRNA [4] and protein [23] expression data were derived from publicly available datasets. All in vitro experiments were performed in at least three biological replicates. Data across biological replicates were calculated and presented as mean values ± SD. GraphPad Prism version 5.01 (GraphPad Software, La Jolla, CA, USA) for Windows was used for dose–response curve modelling and calculation of half maximal effect concentrations (EC50) applying nonlinear regression curve fitting. Treatment groups were compared using One Way ANOVA and subsequent Bonferroni multiple comparisons testing, multiplicity adjusted P values are shown. P values < 0.05 were considered significant. γH2A.X foci distribution histograms were done with GraphPad Prism version 5.01 (GraphPad Software, La Jolla, CA, USA). Heatmap visualizations were generated using MORPHEUS web application (https://software.broadinstitute.org/morpheus).

## Results

### Entinostat treatment of a *MYC*-amplified MB cell line changes the expression of genes involved in targetable pathways

**Fig. 1:**
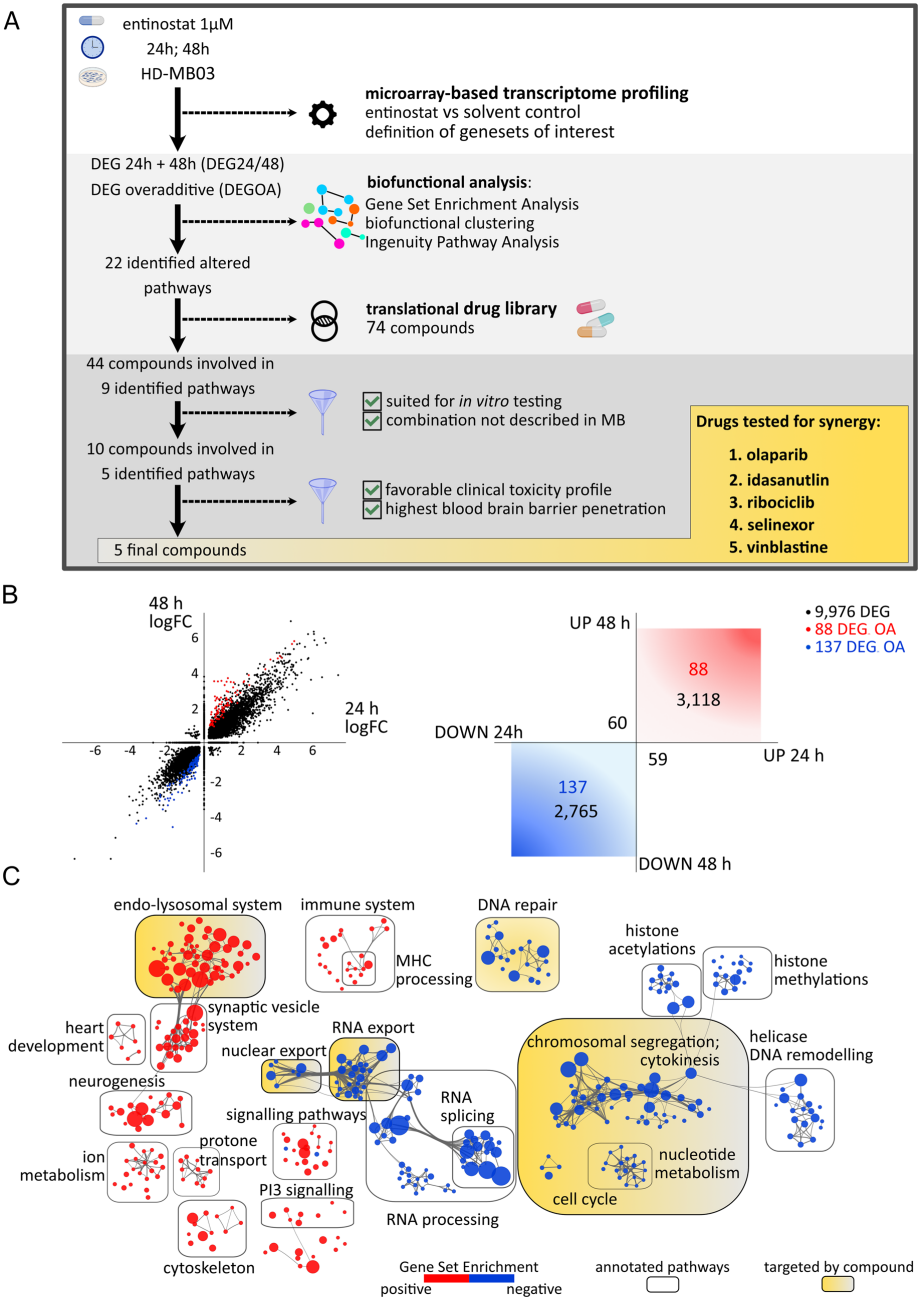
Differential gene expression upon entinostat treatment reveals emerging vulnerabilities for combination treatments. **A** Analysis workflow illustrating the identification of altered pathways under entinostat treatment and the selection of suitable combination-drugs. **B** [left panel] vector graphic illustrating the transcriptomic changes under entinostat treatment. Log_2_FC of DEG with p < 0.05 is shown on x- (24 h treatment value) and y- axis (48 h treatment value). Genes with over-additive behavior shown in red (upregulation) and blue (downregulation); [right panel] sketch graphic with quantification of DEGs. Genes exclusively regulated at one timepoint fall onto the corresponding axis. **C** Results of the GSEA for KEGG, Reactome and GO-Database in DEG_24/48_. Enriched gene sets with FDR < 0.1 are visualized through biofunctional clustering using EnrichmentMap. Positively enriched gene sets shown in red, negatively enriched gene sets in blue. Gene sets with overlap in genes cluster together. Dot size correlates with gene set size. Pathways highlighted in yellow are targeted by the five selected drugs. *FC* fold change, *DEG* differentially expressed genes, *GSEA* gene set enrichment analysis, *FDR* false discovery rate, *OA* over additive

To identify drugs that potentially interact synergistically with entinostat in *MYC*-amplified MB, first, we aimed to identify targetable pathways that were deregulated in response to entinostat single agent treatment (Fig. 1A). The *MY*C-amplified MB cell line HD-MB03 was treated with entinostat and changes in gene expression were determined by microarray-based gene expression profiling after 24 h and 48 h (Fig. 1B). Entinostat treatment led to pronounced changes in gene expression, with 9976 differentially expressed genes (DEGs) across both timepoints (Fig. 1B). The majority of all identified DEGs (n = 6108/9976; 61%) showed consistently either an up- or a downregulation. Of these consistently regulated genes 88 upregulated and 137 downregulated genes were computed to show over-additive behavior i.e. a total of 225 (225/6108 = 4%) DEGs displayed a continuously increasing or decreasing expression over time comparing 24 h and 48 h of treatment (Fig. 1B). A total of 119 (119/6108 = 2%) DEGs differed in their direction of regulation (n = 59 genes first up- then downregulated vs. n = 60 genes first down- then upregulated) comparing both timepoints (Fig. 1B).

We hypothesized that DEGs functionally involved in the previously described cytotoxic effect of entinostat treatment in *MYC*-amplified MB cells [6] are likely to be regulated consistently over time and thus, should be detectable as either up- or down-regulated at both timepoints investigated. In addition, we assumed that deregulated genes showing over-additive change in expression were indicative of a self-sustaining and thus biologically important process. Accordingly, a gene set encompassing all genes differentially regulated at both treatment timepoints (DEG_24/48_) and a gene set of genes showing over-additive behavior comparing both timepoints (DEG_OA_) were defined as gene sets of interest for the subsequent functional analysis (Fig. 1A). GSEA of DEG_24/48_ revealed 1783 significantly (FDR < 0.25) enriched gene sets in the three databases KEGG [24] (n = 141), GO [25] (n = 1360) and REACTOME [26] (n = 282) (Suppl. Table 1). These gene sets clustered into 11 sets of enriched and 11 sets of depleted pathways as evidenced by functional enrichment visualization using cytoscape [17] (Fig. 1C). The respective analysis for DEG_OA_ resulted in 273 significantly enriched gene sets in KEGG (n = 12), GO (n = 68) and REACTOME (n = 193) databases (Suppl. Table 2), clustering into three enriched and nine depleted pathways (Suppl. Figure 1). To strengthen the informative output of this analysis we applied Ingenuity Pathway Analysis (IPA) [18] as a second algorithm to identify functional patterns of deregulated genes. In DEG_24/48_ and DEG_OA_ IPA analysis resulted in 82 and 5 altered canonical pathways respectively (Suppl. Table 3). Accounting for partial functional overlap of identified pathways by GSEA/functional enrichment and IPA analysis we summarized the results by manually grouping all functionally related altered pathways into matching categories. These categories represented a total of n = 22 overarching biological functions and pathways affected by entinostat treatment in HD-MB03 cells (Suppl. Table 4; Fig. 1A). We reasoned that some of the identified pathways may be responsible for entinostat induced cytotoxicity or provide escape mechanisms to evade the cytotoxic stress of entinostat treatment, all of which could represent emerging vulnerabilities. We hypothesized that additional targeting of these pathways may enhance the efficacy of entinostat treatment. To select drugs for testing this hypothesis we compared the list of affected pathways with the mode of action of all drugs included in a published translational drug library (n = 74 drugs). This library includes drugs which are either clinically approved for oncological diseases or under clinical investigation for the treatment of cancers [19] (Suppl. Table 5). Based on literature research we identified n = 44 drugs from this library with known pharmacological interference with one of n = 9 pathways identified as altered upon entinostat treatment (Fig. 1A). We then filtered for drugs with high potential as novel combination partners for entinostat meeting the following two criteria: (1) suited for in vitro testing (leading to exclusion of drugs targeting the immune system); (2) no published data on combination with HDACi in MB (Fig. 1A). In case of two potential drugs targeting the same pathway the compound with highest blood–brain-barrier (BBB) penetrance according to the BBB score [27] was selected for further analysis. As all three vinca alcaloids vinblastine, vincristine and vinorelbine have the same calculated BBB score of 1.19 vinblastine was selected as a drug class representative with favorable clinical tolerability. The following five drugs met all filtering criteria: idasanutlin, olaparib, ribociclib, selinexor and vinblastine (Suppl. Table 6).

### Synergism-screening identifies synergy of PARPi and HDACi in ***MYC*** amplified MB

**Fig. 2:**
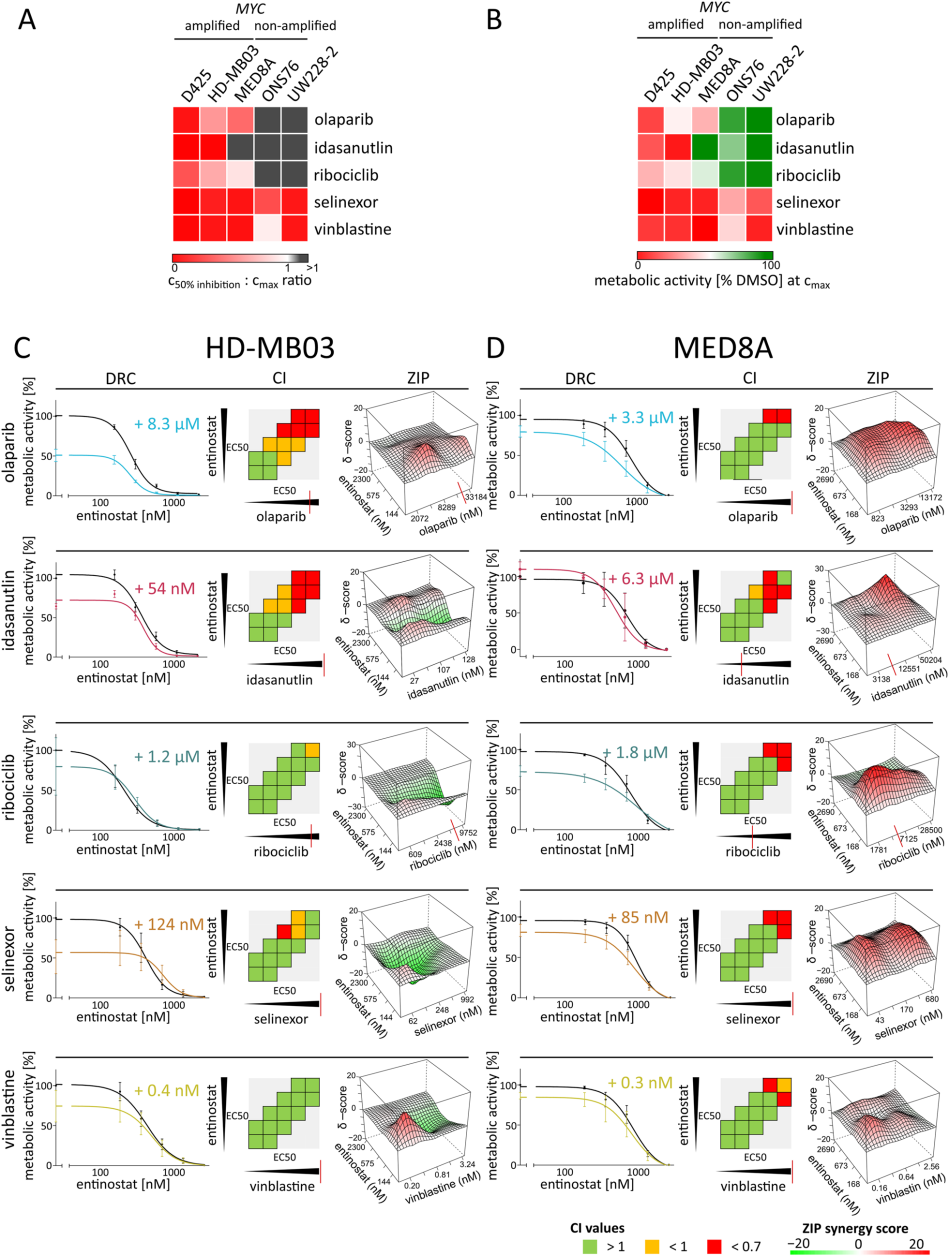
Evaluation of single drug profiles and in vitro synergism-screen in combination with entinostat in *MYC*-amplified models. **A**, **B** Heatmaps comparing single drug profiles in metabolic activity assays with respect to published c_max_ in 3 *MYC*-amplified and 2 non-amplified MB cell lines. **A** absolute IC50 relative to c_max,_. **B** maximum effect achieved at a concentration below the respective c_max_. **C**, **D** Synergism-antagonism analysis for the combination of entinostat with olaparib, idasanutlin, ribociclib, selinexor or vinblastine in HD-MB03 (C) and MED8A (D) for 72 h in a 5 × 5 combination treatment matrix. Left column: dose–response curves of entinostat single treatment (black) and combination with the indicated steady second drug dose. Mean metabolic activity values ± SD relative to DMSO solvent control depicted. Middle column: CI values for 3 constant ratio combinations (diagonal square successions), red: high synergism; orange: synergism; green: antagonism. Right column: ZIP-model based 3D interaction-landscapes computed by SynergyFinder. Red elevations depict synergistic areas with delta-scores indicating percentage of inhibition exceeding expected effect level. Red vertical lines indicate maximal clinically achievable concentration (c_max_). *SD* standard deviation, *DRC* dose response curve shift, *CI* combination index, *ZIP* zero interaction potency

Single agent dose–response curves were determined in metabolic activity assays for all five drugs of interest in three *MYC*-amplified (HD-MB03, MED8A, D425) and two non-*MYC*-amplified MB cell lines (ONS76, UW228-2) (Suppl. Figure 2A and B). To be able to interpret the clinical relevance of the observed in vitro drug potency we calculated the ratio of each drug’s in vitro IC50 and the respective maximal clinical plasma concentration (c_max;_ Suppl. Table 7) (Fig. 2A), as well as each drug’s maximum effect achieved at a concentration below the respective c_max_ (Fig. 2B). Selinexor and vinblastine showed low IC50:c_max_ ratios and profound reduction of metabolic activity across all cell lines, indicating a strong overall cytotoxicity (Fig. 2A, B). Only the PARP inhibitor (PARPi) olaparib showed a low IC50:c_max_ ratio (Fig. 2A) as well as a strong inhibition of metabolic activity at concentrations below c_max_ (Fig. 2B) exclusively in all three *MYC*-amplified cell lines, suggesting both a translationally relevant single agent activity and a *MYC* dependent drug response pattern.

All five compounds were tested for synergistic interaction with entinostat in two *MYC*-amplified cell lines (HD-MB03 and MED8A) (Fig. 2C, D). We evaluated synergism applying three different methods to increase reliability: I) dose–response-curve (DRC) shift-analysis; II) Chou-Talalay combination index (CI) method; III) response-surface ZIP synergy computation (Fig. 2C, D; left to right). The most robust indication for synergism was observed for the combination of entinostat and olaparib showing synergistic interaction in all three synergy models in HD-MB03, and in two of three models (DRC and ZIP) in MED8A (Fig. 2C, D). Importantly, in both cell lines indications for synergism were observed at clinically achievable concentrations (Suppl. Table S7).

### Synergistic interaction of PARPi and entinostat is a drug class effect in ***MYC***-amplified MB

**Fig. 3:**
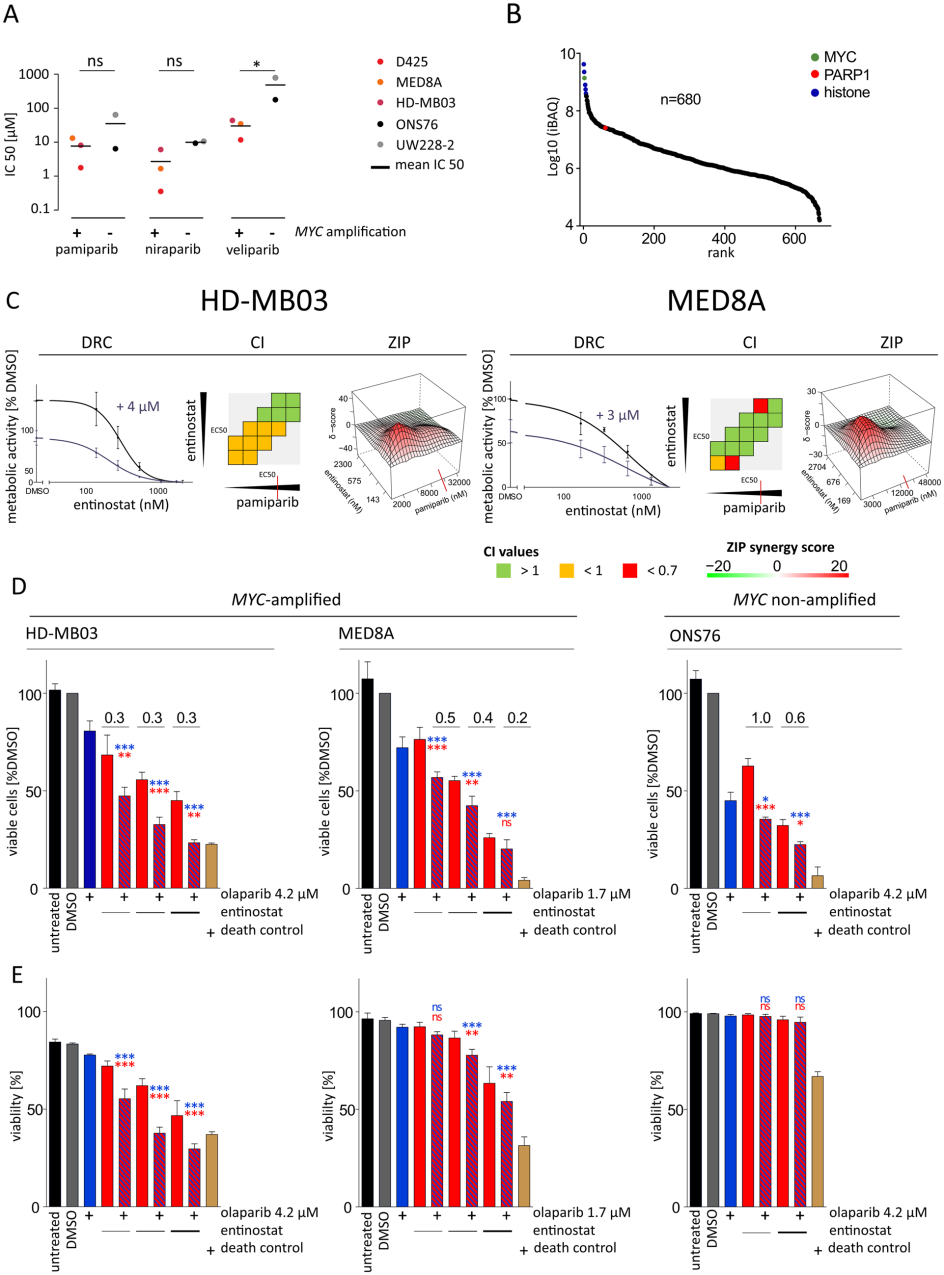
Validation of entinostat and PARPi as synergistic drug partners. **A** IC50 values (dots) of *MYC*-amplified (left, colored) and non-*MYC*-amplified MB cell lines (right, grey scale) for pamiparib, niraparib, veliparib. *p < 0.05, ^ns^ not significant (two tailed t-test) **B** Data analysis of mass spectrometry (MS) after pulldown of endogenous MYC in HD-MB03 cells with detection of n = 680 proteins revealing colocalization with PARP1. **C** Synergism-antagonism analysis for the combination treatment of entinostat with pamiparib in HD-MB03 (left) and MED8A (right) for 72 h in a 5 × 5 combination treatment matrix. Left column: dose–response curves of entinostat single treatment (black) and combination with the indicated pamiparib concentration. Mean metabolic activity values ± SD relative to DMSO solvent control depicted. Middle column: CI values for three constant ratio combinations (diagonal square successions), red: high synergism; orange: synergism; green: antagonism. Right column: ZIP-model based 3D interaction-landscapes computed by SynergyFinder. Red elevations depict synergistic areas with delta-scores indicating percentage of inhibition exceeding expected effect level. Red vertical lines indicate maximal clinically achievable concentration (c_max_). **D** and **E** cell number (top) and viability (bottom) reduction after 72 h single (plain-colored bars) and combination (striped bars) treatment with entinostat (red) and olaparib (blue) in two *MYC*-amplified (left and middle; HD-MB03 and MED8A) and one non-amplified (right; ONS76) MB cell line determined by automated cell counting of trypan blue-exclusion assay. Entinostat treatment was applied in a twofold dilution series for MED8A and HD-MB03 including previously determined metabolic activity IC50 values (HD-MB03: 575 nM; MED8A: 672.5 nM). Treatment concentrations in ONS76 same as in HD-MB03. 500 nM staurosporine served as death control. Bars depict mean values ± SD of three independent replicates. Combination index (CI) values indicated above black lines indicating compared columns, CI < 1 indicates synergism, CI = 1 additivity and CI > 1 antagonism. p < 0.05, **p < 0.01; ***p < 0.001; ^ns^ not significant (one way ANOVA with Bonferroni's Multiple Comparison Test); *iBAQ* intensity Based Absolute Quantification; *DRC* dose response curve shift, *CI* combination index, *ZIP* zero interaction potency

To confirm the sensitivity of *MYC-*amplified MB cells to single agent olaparib treatment to be a drug class effect the response of MB cells to three additional PARPis (niraparib, pamiparib, veliparib) was evaluated (Fig. 3A, Suppl. Figure 2C). All applied PARPis bind to the catalytic domain of PARP but differ in their PARP trapping ability, off-target kinase affinities, pharmacokinetic profiles and reported BBB penetration [28–30]. *MYC-*amplified cell lines showed a trend towards or a significantly higher sensitivity to all three PARPis compared to non-*MYC-*amplified cell lines (Fig. 3A, Suppl. Figure 2C). Of note, significantly higher sensitivity of *MYC*-amplified cell lines was observed for veliparib, which has the lowest reported PARP-trapping activity [30] (Fig. 3A). The increased sensitivity of *MYC*-amplified MB cells to all applied PARPis raised the question whether there is functional interdependence of PARP and MYC protein. Interestingly, the analysis of previously published mass spectrometry data of protein pulldown after immunoprecipitation of endogenous MYC in HD-MB03 cells [9] revealed PARP1 peptides co-localizing with MYC ranking in the top 10^th^ percentile of all MYC-interacting peptides, suggesting a protein–protein interaction of MYC and PARP1 (Fig. 3B). Synergistic interaction of entinostat and PARPi as a drug class effect was validated with pamiparib, showing synergistic interaction with entinostat in both *MYC*-amplified cell lines tested (HD-MB03 and MED8A) in 2/3 models (DRC and ZIP) (Fig. 3C). Notably, for both olaparib and pamiparib, the synergistic interaction was observed within the range of clinically achievable plasma concentrations (Suppl. Table S7).

Continuing our investigation with the FDA-approved compound olaparib we aimed to validate the observed synergistic interaction in a different experimental setting. We performed cell count experiments showing a significant, dose-dependent reduction of the number of viable cells upon combination treatment in MED8A and HD-MB03 with computed combination indices below 0.5, indicating strong synergy for all tested conditions (Fig. 3D). Also in the non-*MYC*-amplified cell line ONS76 the number of viable cells was significantly reduced upon combination treatment with a combination index indicating synergy in one of two tested conditions (Fig. 3D). Conversely, cell viability was significantly reduced only in the *MYC*-amplified cell lines (Fig. 3E), indicating increased cell death as opposed to a possible cell cycle arrest in non-*MYC*-amplified cells.

### ***MYC***-amplified MB cells show reduced DDR capacity upon entinostat treatment, and the target PARP is expressed in primary ***MYC***-driven MB

**Fig. 4:**
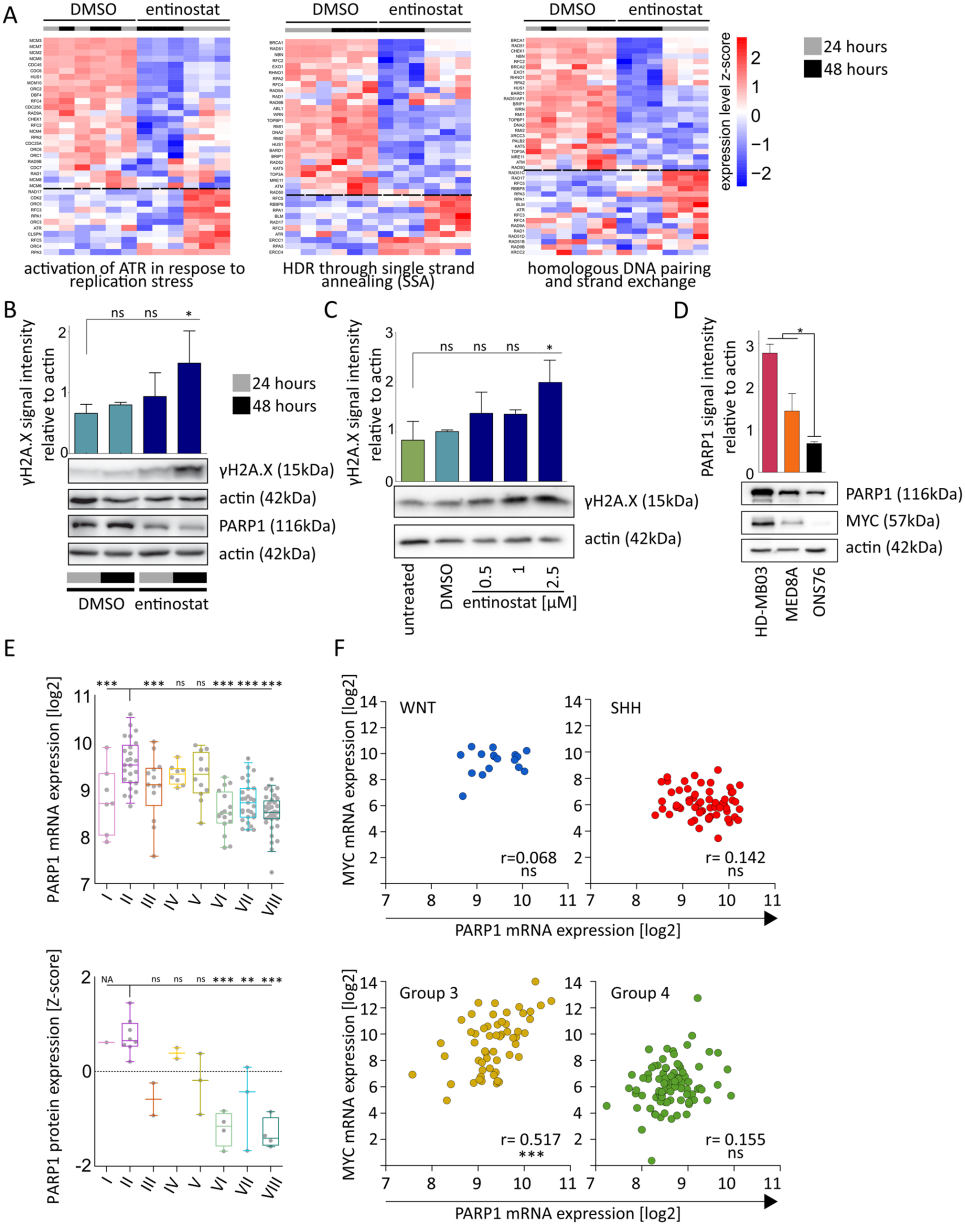
Combination treatment with entinostat and olaparib significantly decreases cell growth and cell viability and induces sub-G1 fractions in *MYC*-amplified MB cell lines. **A** Heatmaps of ranked genes according to expression differences (z-score) between the two phenotypes (DMSO vs. 1 µM entinostat) of three representative DDR-related gene sets negatively enriched in DEG_OA_ with lowest FDR displayed for the three biological replicates of 1 µM entinostat treatment for 24 h and 48 h in HD-MB03. Genes ranked above the dashed line show decreased expression under entinostat treatment. **B** Western blot analysis for PARP1 and γH2A.X in GEP matched protein samples of 1 µM entinostat treatment in HD-MB03, bar graph depicts densitometric analysis of γH2A.X signal intensity relative to actin in three biological replicates. **C** Western blot analysis of γH2A.X increment in HD-MB03 cells treated for 24 h with indicated concentrations of entinostat, bar graph depicts densitometric analysis of γH2A.X signal intensity relative to actin in three biological replicates. **D** Western blot analysis of basal PARP1 and MYC protein levels MB cell lines developed on same blot, actin serves as loading control, bar graph depicts densitometric analysis of PARP1 signal intensity relative to actin in three biological replicates. *p < 0.05, ^ns^ not significant (one-way ANOVA with Bonferroni's Multiple Comparison Test) **E**: Box-dot plot comparing PARP1 mRNA (upper panel) and protein (lower panel) expression in primary MB group3/4 subtypes. Dots represent single samples. Boxes extend from 25 to 75th percentiles, whiskers extend from 5-95^th^ percentiles, lines at median. *p < 0.05, **p < 0.01; ***p < 0.001; ^ns^ not significant (one-way ANOVA with Bonferroni's Multiple Comparison Test). **F** Correlation of *PARP1* and *cMYC* mRNA expression in Wnt- (blue), SHH-(red), Group 3 (yellow), Group 4 (green) primary MB data. Pearson product-moment correlation coefficient (r) was computed using GraphPad Prism 5. ***p < 0.001; ^ns^ not significant (two tailed t-test). mRNA expression data was derived from R2 (3)

We hypothesized that the observed synergy of entinostat and olaparib is functionally based on a joint inhibiting effect on the DNA damage response (DDR) leading to increased and potentially fatal accumulation of DNA damage. Indeed, we observed a strong overrepresentation of downregulated DDR gene sets (n = 4, see exemplary sets in Fig. 4A) in the top ranked gene sets with an FDR < 0.01 (n = 25) considering all genes with a time dependent over-additive change in expression upon entinostat treatment (DEG_OA_) (Suppl. Table 2). Based on this observation we reasoned that entinostat may exert its effect on DDR indirectly by affecting the transcription of genes involved in the DNA damage repair machinery. In line with this observation, we detected a time- (Fig. 4B) and dose-dependent (Fig. 4C) increase in phosphorylated histone variant γH2A.X, an early marker of DNA double strand breaks (DSBs), upon entinostat single agent treatment. Taken together these data point towards a reduced DNA damage repair capacity of HD-MB03 cells upon entinostat treatment.

The role of PARP1 as a crucial initiator of DDR is well described [31]. To verify that olaparib can exert its described effect by inhibiting PARP1 in our model systems we performed target presence analysis. In all three cell lines we confirmed PARP1 protein expression with significantly higher PARP1 expression in the *MYC*-amplified cell lines (Fig. 4D). *PARP1* expression was not significantly altered upon entinostat treatment in HD-MB03 cells on mRNA level (Suppl. Table 8: *PARP1* unlisted). On protein level we observed significantly reduced but readily detectable amounts of PARP1 protein after entinostat treatment, (Fig. 4B, Suppl. Figure 3A). We confirmed target presence in primary MB tumors by analyzing publicly available mRNA [4] and protein [23] expression data sets. *PARP1* showed strong expression on both mRNA (Fig. 4E top) and protein level (Fig. 4E bottom) in primary *MYC*-amplified MB tumors of subtype II with a trend towards or significantly higher expression compared to all other subtypes. Additionally, PARP1 expression significantly correlated with *MYC* expression on mRNA level in Group 3 tumors (Fig. 4F). *PARP2* expression levels did not differ significantly between Group 3/4 subgroups I-VIII (Suppl. Figure 3B).

We conclude that PARP1 is abundantly expressed in *MYC-*driven MB and that our cell culture models are suitable to study joint effects of entinostat and olaparib treatment on DDR.

### Entinostat treatment leads to increased DNA damage accumulation upon treatment with olaparib and DNA damaging chemotherapeutics in *MYC*-amplified cell lines

**Fig. 5:**
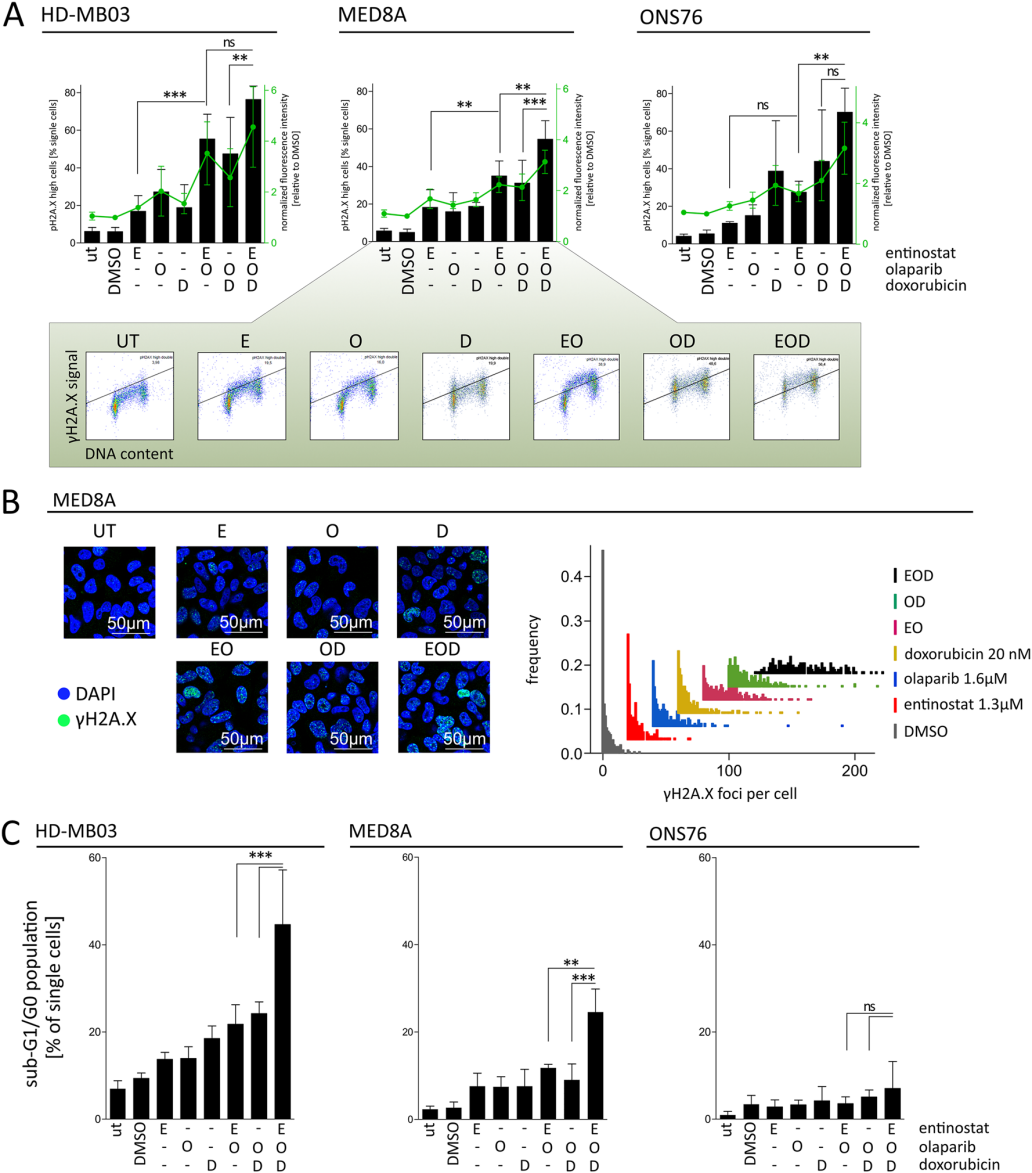

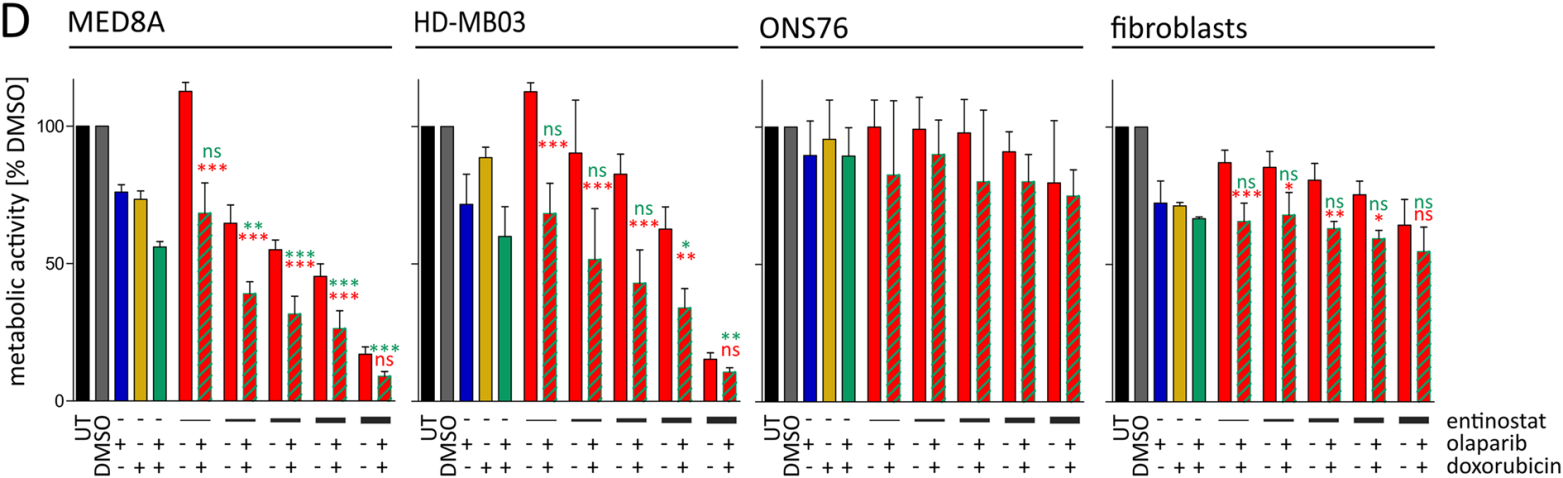
Early formation of DNA DSBs precedes cell death induction in *MYC*-amplified MB cell lines. **A** DNA DSB formation in HD-MB03, MED8A, and ONS76 after 24 h treatment. γH2A.X positive cells (black bars) and FC in mean normalized fluorescence intensity (green line). Depicted are mean values ± SD of at least three independent replicates; *p < 0.05, **p < 0.01; ***p < 0.001; ^ns^ not significant (one way ANOVA). lower panel: representative bivariate scatterplot of flow cytometric analysis of PI and γH2A.X co-staining. PI signal intensity displayed on x-axis, Alexa-488 fluorescence intensity (γH2A.X) on y-axis. **B** [left] representative confocal immunofluorescence microscopy images of γH2A.X staining in MED8A after 24 h treatment. Nuclei are counterstained with DAPI. [right] representative frequency distribution analysis of γH2A.X foci per cell in MED8A. **C** SubG1/G0 cell cycle population analysis after 72 h entinostat (E), olaparib (O), doxorubicin (D) single and combination treatments in two *MYC*-amplified (HD-MB03 and MED8A) and one non-amplified (ONS76) MB cell line determined by flow cytometric assessment of propidium iodide DNA staining. Treatment concentrations for HD-MB03/MED8A: 575 nM/1345 nM Entinostat (E); 50 nM/20 nM Doxorubicin (D); 4.2 µM/1.7 µM Olaparib (O). ONS76 treatment concentrations same as HD-MB03. Fractions of total single cells detected in sub-G1/G0 phase displayed at each treatment condition. Bars depict mean values ± SD of at least three independent replicates. *p < 0.05, **p < 0.01; ***p < 0.001; ^ns^ not significant (one way ANOVA). D: metabolic activity after 72 h olaparib- (O; blue bars), doxorubicin (D; yellow bars) single, olaparib-doxorubicin combination (OD; green bars), increasing concentrations of entinostat (E; red bars) and triple combination treatments (striped bars). Doxorubicin concentration: 10 nM. Olaparib concentrations: 4.1 µM (HD-MB03, ONS76, fibroblasts); 1.7 µM (MED8A). Entinostat concentrations: 250 nM/500 nM/750 nM/1 µM/2 µM in MED8A/HD-MB03; 1 µM/1.5 µM/2 µM/5 µM/10 µM in ONS76/fibroblasts. Significance levels were computed in comparison to entinostat single treatment (red asterisks) and olaparib-doxorubicin combination treatment (green asterisks) *p < 0.05, **p < 0.01; ***p < 0.001; ns not significant (one way ANOVA). *UT* untreated

It has been previously shown that the cytotoxic effects of olaparib can be increased by concomitant treatment with DNA-damaging agents such as doxorubicin [32]. To test the impact of a DNA damaging agent in the presence of entinostat and olaparib we quantified the number of DNA DSBs by staining of the phosphorylated histone variant γH2A.X after 24 h of treatment with entinostat, olaparib and doxorubicin in single, double and triple drug treatment. Flow cytometry analysis revealed that single treatments generated comparable amounts of DSBs across all cell lines tested (Fig. 5A). The combination of entinostat and olaparib lead to a significantly increased number of DSBs compared to entinostat single agent treatment only in the *MYC*-amplified cell lines (Fig. 5A). Similarly, and in line with our hypothesis, addition of entinostat to the combination of olaparib and doxorubicin (OD) significantly enhanced the number of DSB formation only in the *MYC-*amplified cell lines (Fig. 5A). Cell cycle analysis by PI staining revealed that olaparib treated cells showed highest γH2A.X positivity in S-phase (Suppl. Figure 3C), in line with DSBs caused by PARP-trapping-induced replication fork collapse. Entinostat and doxorubicin single treatment did not show a cell cycle specific distribution of DSBs (Suppl. Figure 3C). Interestingly, upon triple treatment the *MYC* non-amplified cell line ONS76 showed strongest accumulation of DNA damage in G2/M phase, while the *MYC*-amplified cell lines showed the lowest number of DNA damage in G2/M phase (Suppl. Figure 3C). Comparing the number of cells without (no γH2A.X foci per cell) and with strong accumulation (≥ 10 γH2A.X foci per cell) of DNA damage revealed a significant increase of cells with ≥ 10 γH2A.X foci per cell upon triple treatment compared to DMSO control in *MYC*-amplified MED8A cells (Suppl. Figure 3D). Additionally there were significantly less cells without any γH2A.X focus in double and triple treatment conditions compared to DMSO control (Suppl. Figure 3D). In summary, we observed strong induction of DSBs upon triple treatment specifically in *MYC*-amplified MB cell lines with preferential occurrence during G1/S phases.

We assumed that accumulation of DNA damage, particularly during G1/S phases may lead to increased cell death. Indeed, we observed slightly increased sub-G1 fractions compared to single agent treatment in all cell lines after 72 h of double treatments. Importantly a significant increase of the sub-G1 fraction was detected exclusively in *MYC*-amplified cell lines upon triple combination treatment (Fig. 5C; Suppl. Figure 4A, B). Conversely, the *MYC* non-amplified cell line ONS76 showed a G2/M arrest upon treatment (Suppl. Figure 4A, B), which is in line with the preferential occurrence of DSBs during G2/M cell cycle phase. Finally, addition of entinostat (E) to a double treatment of olaparib and doxorubicin (OD) led to a significant decrease in metabolic activity compared to backbone treatment (OD) alone only in *MYC*-amplified MB cells (Fig. 5D). Notably also in non-malignant non-transformed juvenile fibroblasts the triple treatment (EOD) did not cause significantly lower metabolic activity in comparison to single agent treatment with olaparib and doxorubicin (Fig. 5D).

Taken together we observed formation of DSBs as an early response to the triple combination treatment preceding the induction of cell death specifically in *MYC*-amplified MB cell lines.

## Discussion

Promising advances in the genomic, epigenomic, and proteomic characterization of *MYC*-driven MB have not yet resulted in biomarker-guided targeted treatment regimens. Patients with group 3 *MYC*-driven MBs face a very dismal prognosis and novel therapeutic approaches are urgently needed. Here, we analyzed the effect of entinostat treatment on the transcription profile of *MYC*-driven MB cells to better understand why these cells are highly susceptible to class I HDACi treatment and to deduce potential drug partners for synergistic or synthetic lethal combination therapies.

The majority of pathways that were affected by entinostat treatment in our data (DDR, cell cycle, RNA processing, differentiation) has previously been reported to be affected by HDACi also in other cancer entities [33, 34], suggesting that some of these effects may be rather drug class (HDACi) than entity specific. In line with previous findings [35] we also confirmed the enrichment of immunological pathways and upregulation of HLA class I heavy chain paralogues upon entinostat treatment. This supports the role for class I HDACis in immune cancer therapies, which is currently under clinical investigation in several clinical trials [36] (including in pediatric oncology, see for example NCT03838042; nivolumab/entinostat).

Our synergy experiments identified synergism of entinostat and PARPis in *MYC*-amplified MB cells lines at translationally relevant drug concentrations with regard to c_max_ reported in patients. Of note, we encountered previously described synergism model limitations in form of inconsistencies between applied synergism computations, leading us to integrate distinct models and emphasizing the importance of synergy validation experiments [37].

We hypothesize that the functional explanation for the observed synergy of olaparib and entinostat is a joint effect on a cell’s DNA damage repair capacity, suggesting a drastically reduced DNA damage repair upon combination treatment. This is in line with our observation of increased levels of DSBs upon combination treatment, which could be dramatically increased by additional treatment with the DNA damaging agent doxorubicin. While PARP inhibition directly leads to reduced DNA damage detection signaling [38], HDAC inhibition treatment has been described to impair NHEJ- or HR-repair by transcriptional repression of involved genes including *ATR*, *ATM*, *BRCA1*, *BRCA2*, *Ku70*, *Ku80*, *DNA-PK*, *ligase IV*, *XRCC4*, *RAD51*, *CHEK1* and *CHEK2* [33, 39]. The expression of all of these genes was also affected by entinostat treatment in our data. Considering this highly consistent deregulation of DDR genes upon HDACi treatment, we assume that also other, clinically less advanced compounds interfering with DDR such as CHEK1/2 kinase inhibitors, DNA-PK inhibitors or ATR inhibitors may be promising combination partners for entinostat in this context.

Synergistic interaction of HDACi and PARPi has been previously described in vitro and in vivo including the combination of vorinostat/olaparib in GBM cell lines [40], entinostat/olaparib in HR-proficient ovarian cancer cells [39], as well vorinostat/veliparib, vorinostat/olaparib and belinostat/olaparib in triple negative breast cancer cells and xenograft models [41, 42]. Three clinical trials are currently investigating this drug class combination (NCT03924245; NCT03742245; NCT04703920) in gynecological entities.

Notably, in our data *MYC*-amplified MB models showed increased susceptibility to both single agent and combination treatment compared to non-*MYC* amplified MB cells. Investigation of gene expression and protein expression data of primary MB confirmed high PARP1 expression and correlation with *MYC* expression, particularly in subtype II MB samples. This is in line with previously published data showing elevated PARP1 expression in MB correlating with poor disease outcome [43]. We hypothesize that MB cells with high *MYC* expression are prone to acquire DNA damage caused by MYC-mediated replication stress, which may explain why *MYC*-amplified MB cells are particularly dependent on a functioning DNA damage signaling and repair machinery. This is in line with previously published data showing increased sensitivity of cancer cells with high *MYC/MYCN-*expression to PARPi including glioblastoma stem-like cells, neuroblastoma, multiple myeloma and medulloblastoma cells in vivo and in vitro [44–47]. The data provided here on a possible co-localization of MYC and PARP1 has not been previously described and warrants further investigation since it may provide further insight into a mechanistic interaction of both proteins. The factor of potential underlying BRCAness, as reported previously in primary group 3 MB samples, might also play a role in the observed sensitivity to PARPi [4, 48].

Our data suggest that additional induction of DNA damage (i.e. with doxorubicin) may be beneficial to fully exploit the reduced DDR capacity of cells treated with entinostat and olaparib. The combination of PARPi with a DNA-damaging chemotherapy is already being investigated in adult (NCT03161132; NCT00740805) and pediatric clinical trials (NCT02813135; NCT03749187; NCT02044120) [32, 49]. Importantly in the field of neuro-oncology several trials investigate PARPi treatment in combination with radiotherapy and/or temozolomide in HGG (NCT03581292; NCT03212742; NCT03749187).

However, the combination of a PARPi plus a DNA damaging chemotherapeutic agent shows important and dose limiting myelotoxicity in the clinic [50]. Accordingly, the identification of predictive biomarkers to select patients that are most likely to benefit from such a treatment, possibly at comparably low and thus better tolerable concentrations is crucial for a successful translation into the clinic. Our data suggests that entinostat might confer selectivity of DNA damage accumulation in *MYC*-driven MB cells and reduce DNA damage tolerance selectively in transformed cells, thereby overcoming previously reported normal tissue toxicities [50]. Further studies are needed to investigate the toxicity profile of this triple drug combination in in vivo models.

## Conclusion

In summary, our data provide evidence for a synergistic interaction of class I HDACi entinostat and PARPis in *MYC*-amplified MB cell lines inducing increased sensitivity to the DNA damaging agent doxorubicin. Further in vivo investigations are warranted to explore the value of MYC as a predictive biomarker and to explore the translational potential of this novel drug combination for the treatment of patients with *MYC*-amplified MB.

### Supplementary Information

Below is the link to the electronic supplementary material.

Supplementary file1 (PDF 314 KB)
Figure S1 EnrichmentMap for of GSEA of DEGOA. Results of the GSEA for KEGG, Reactome and GO-Database in DEGOA. Enriched gene sets with FDR < 0.1 are visualized through biofunctional clustering using EnrichmentMap. Positively enriched gene sets shown in red, negatively enriched gene sets in blue. Gene sets with overlap in genes cluster together. Dot size correlates with gene set size. Biomechanisms highlighted in yellow are targeted by the final five compounds.
Figure S2. Single treatment dose response profiles of idasanutlin, olaparib, selinexor, ribociclib, vinblastine and the PARPi niraparib, pamiparib, veliparib in MB cell lines. A: Single dose-response curves of metabolic activity read-out after 72h treatment with idasanutlin, olaparib, ribociclib, selinexor, vinblastine in MYC-amplified (warm colors) and non-MYC-amplified MB cell lines (grey shades). Depicted are mean values ± SD relative to DMSO solvent control. Relative IC50 values of each drug in the corresponding cell line are listed in grey boxes. B: Comparison of IC50 values (dots, left y-axis) between MYC-amplified (left) and non-MYC-amplified MB cell lines (right) for idasanutlin, olaparib, selinexor, ribociclib and vinblastine. C: Single dose-response curves of metabolic activity read-out after 72h treatment with and niraparib, pamiparib, veliparib, legend and statistics as in (A)
Figure S3 Quantification of DNA damage by γH2A.X staining. A: Bar graph depicts densitometric analysis of PARP1 signal intensity relative to actin in three biological replicates of western blot shown in Fig. 4B Significance calculated comparing each column to the 24h untreated condition, *p < 0.05, ns = not significant, (one way ANOVA). B: Box-dot plot comparing PARP2 mRNA expression in primary MB group3/4 subtypes. Dots represent single samples. Boxes extend from 25-75th percentiles, whiskers extend from 5-95th percentiles, lines at median. *p < 0.05, **p < 0.01; ***p < 0.001; ns not significant (one-way ANOVA with Bonferroni's Multiple Comparison Test). C: Visualization of mean FC in normalized γH2A.X fluorescence intensity separately for cell cycle phases of cycling cells compared to DMSO control after 24h single treatment with Entinostat, Olaparib, Doxorubicin and the respective triple combination treatment depicted as heat map. Treatment concentrations correspond to Fig. 5A. Mean FC values are displayed in the respective squares and were calculated for at least three independent replicates. D: Bar graph depicting the percentage of cells without γH2A.X foci (green) and with more than 10 γH2A.X foci per cell (black) in MED8A cells at the indicated treatment conditions determined by immunofluorescence microscopy. Experiments are performed in biological triplicates. Significance calculated comparing each column to the respective DMSO control, *p < 0.05, **p < 0.01; (one way ANOVA).
Figure S4 Integral cell cycle profile analysis for entinostat, olaparib and doxorubicin single-, double-, and triple treatment at 72h. A: Cell cycle distribution of sub-G1/G0- , G1-, S-, and G2M-population in % of single cells measured after 72h treatment with entinostat (E), olaparib (O), doxorubicin (D) and the respective combinations. Treatment concentrations correspond to Fig. 5C. Bars depict mean values ± SD of at least three independent replicates. B: Visualization of mean FC in cell cycle population compared to DMSO control after 72h single treatment with E, O, D and the respective triple combination treatment depicted as heat map. Treatment concentrations correspond to Fig. 5C. Mean FC values are displayed in the respective squares and were calculated for at least three independent replicates.

Supplementary file2 (XLSX 101 KB)

Supplementary file3 (XLSX 25 KB)

Supplementary file4 (XLSX 12 KB)

Supplementary file5 (XLSX 13 KB)

Supplementary file6 (XLSX 9 KB)

Supplementary file7 (XLSX 10 KB)

Supplementary file8 (XLSX 10 KB)

Supplementary file9 (XLSX 560 KB)

## Data Availability

The datasets generated and analyzed during the current study are available in the GEO repository under GSE accession number GSE232148.
