# Supplementary figures and images for: Class I HDAC inhibition reduces DNA damage repair capacity of MYC-amplified medulloblastoma cells
Source: J Neurooncol. 2023 Oct 3;164(3):617–32. doi: 10.1007/s11060-023-04445-w (PMC10589189; doi:10.1007/s11060-023-04445-w)

Figure S1

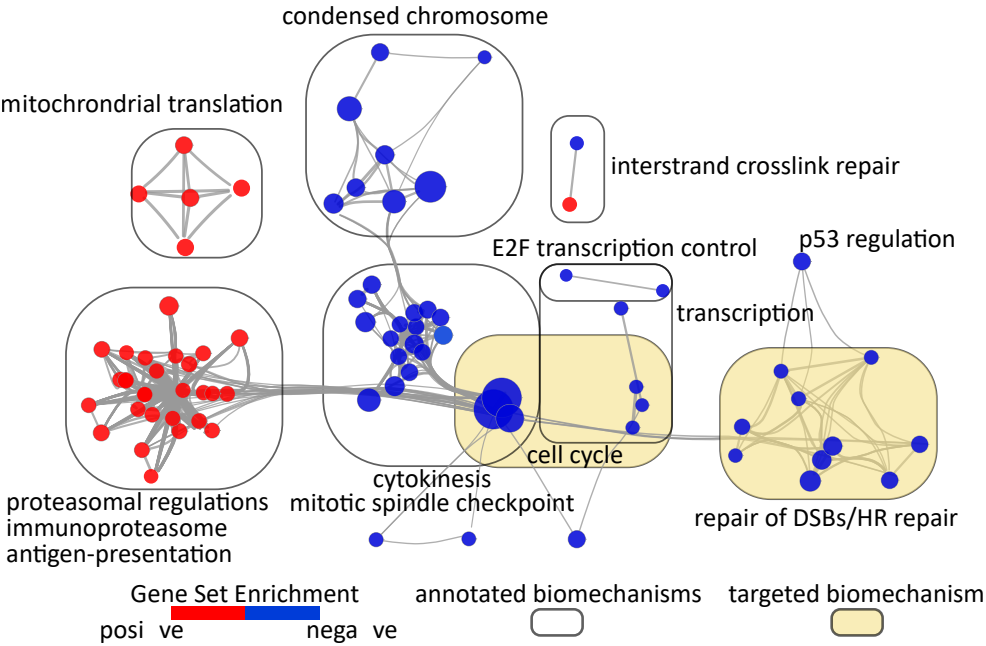

Figure S2  
A

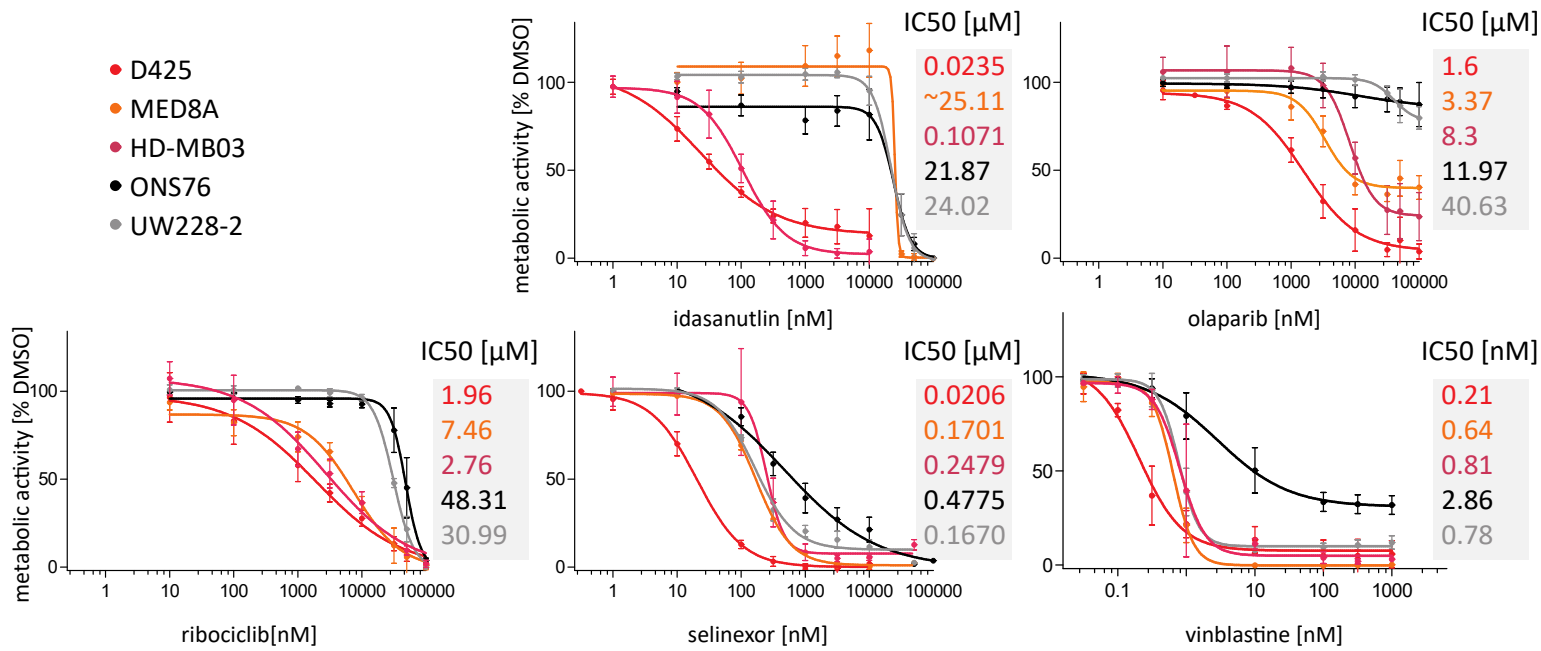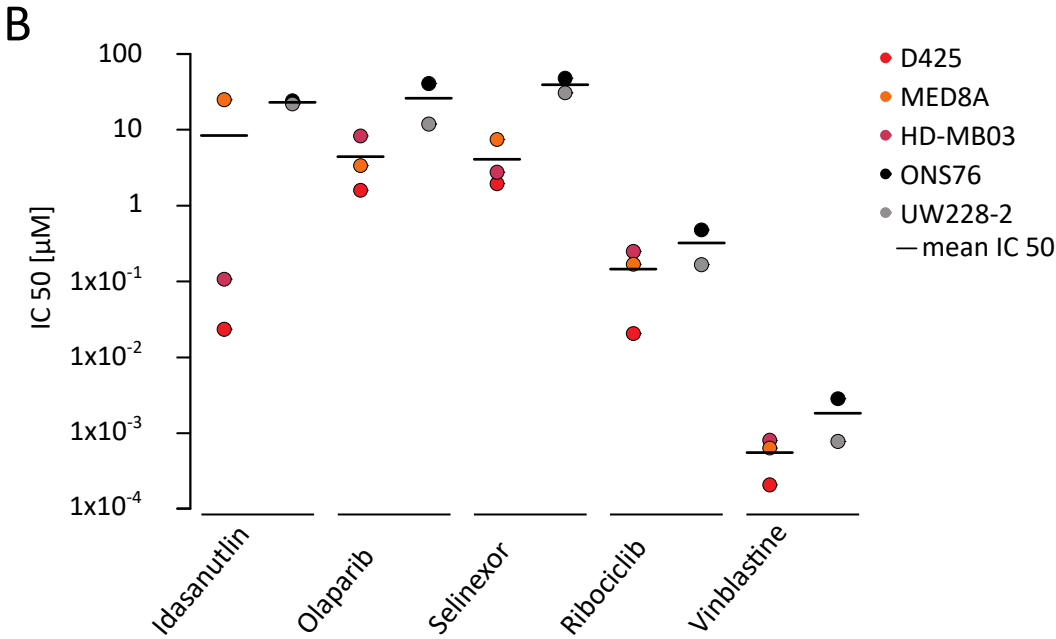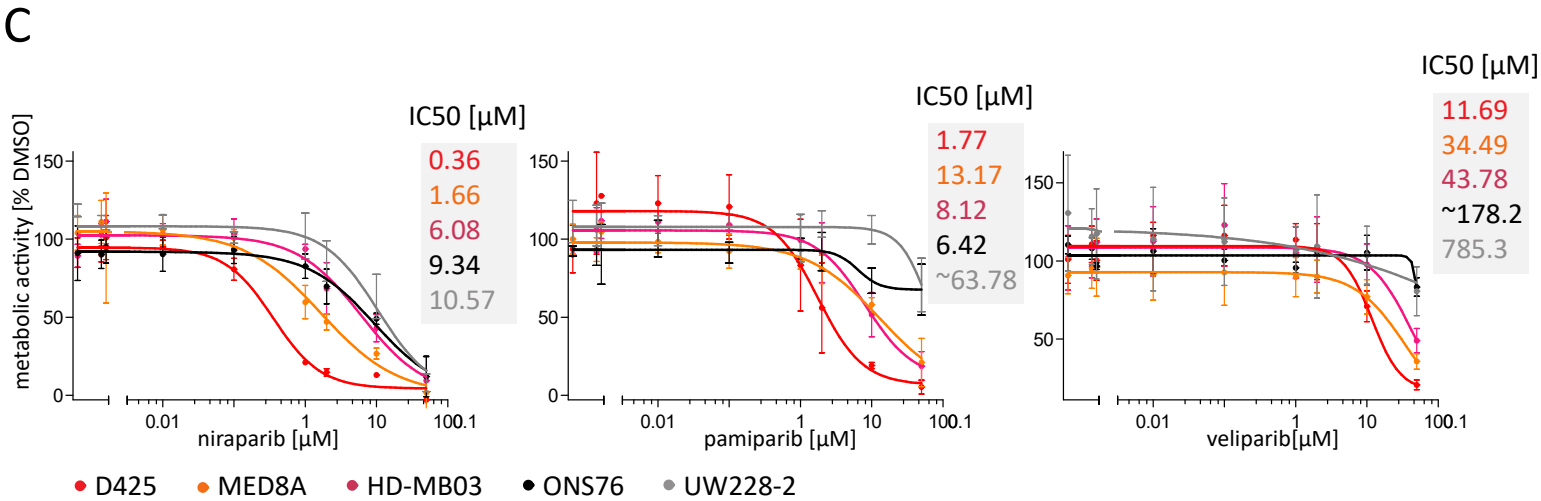

Figure S3

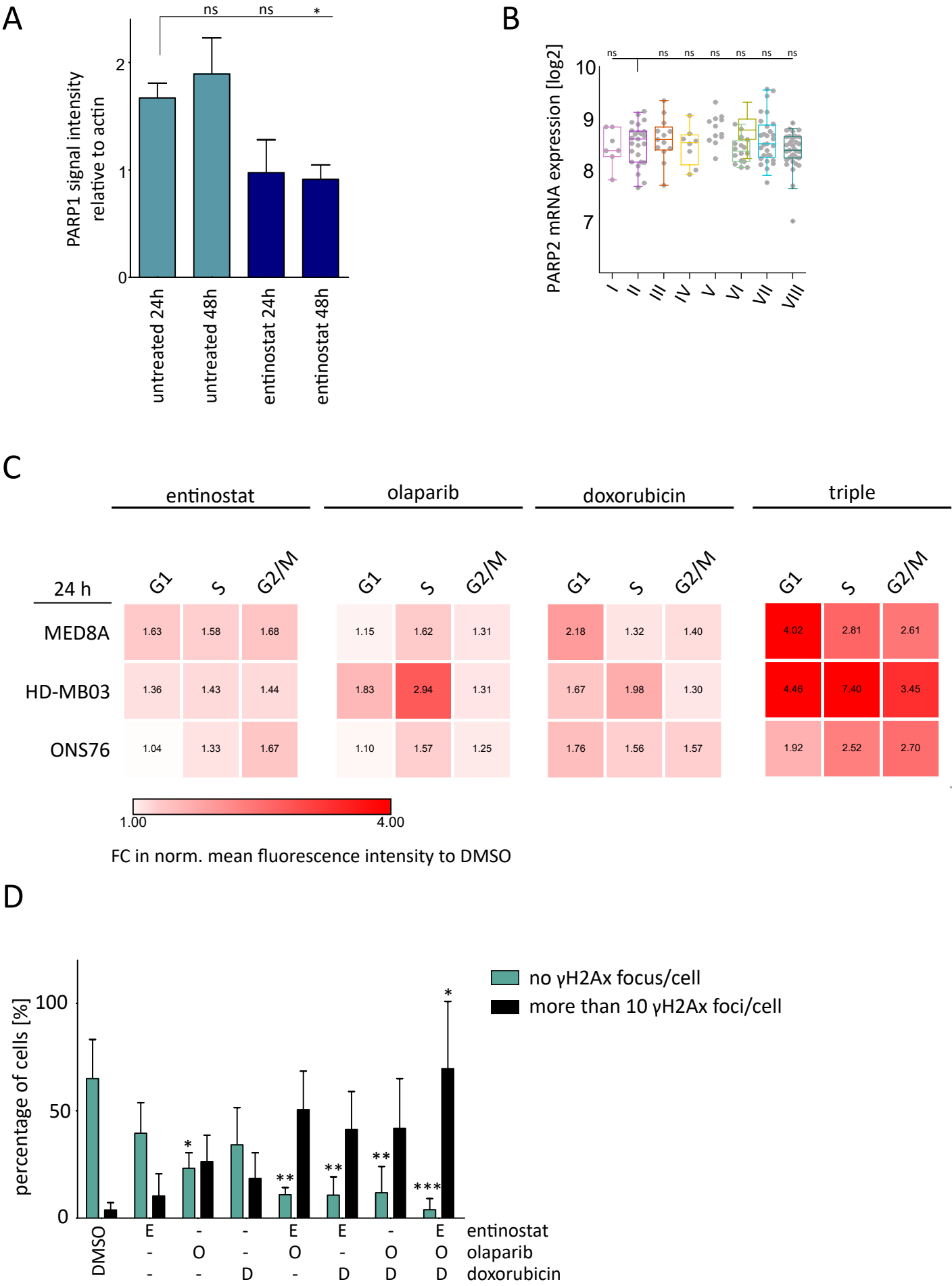

Figure S4

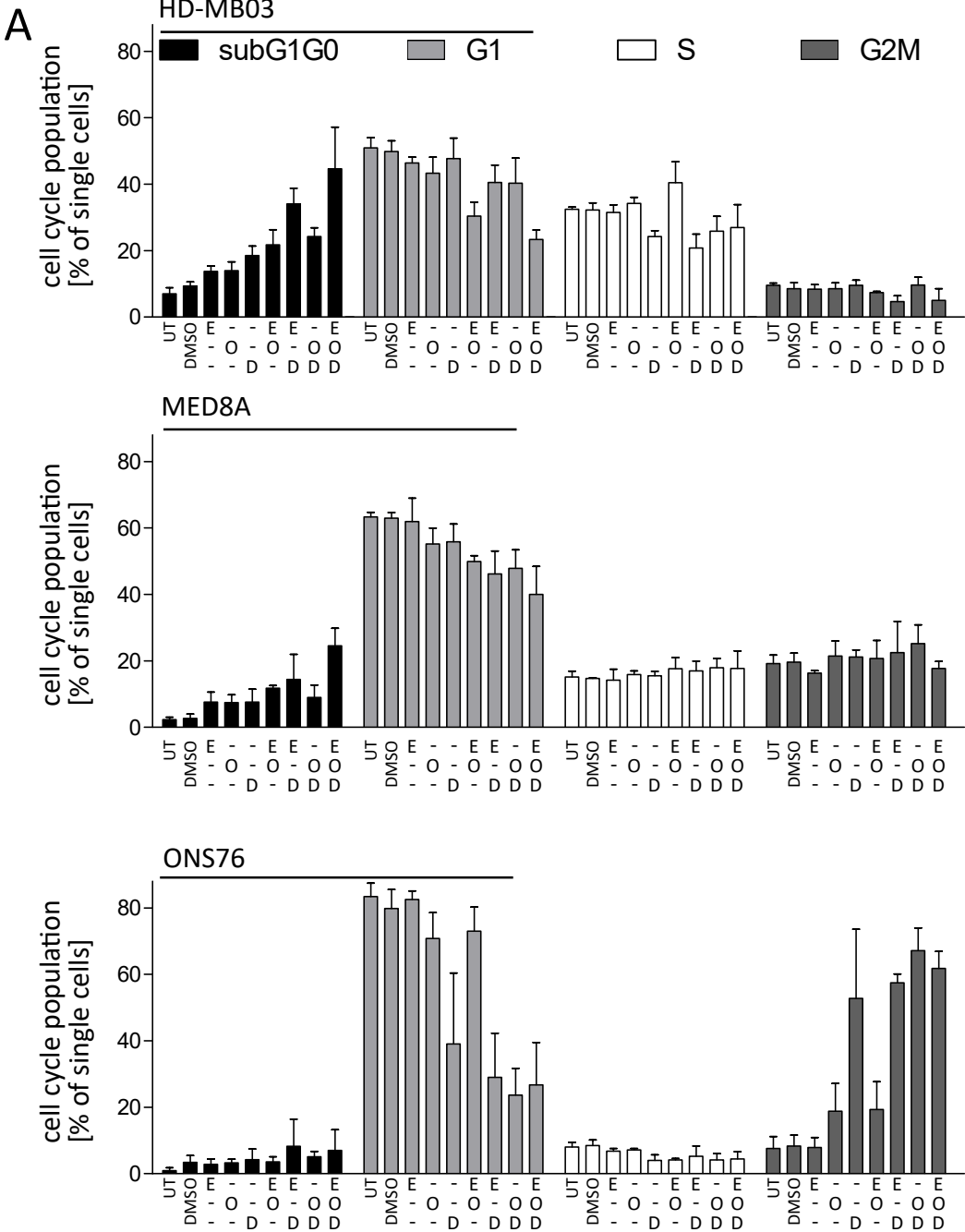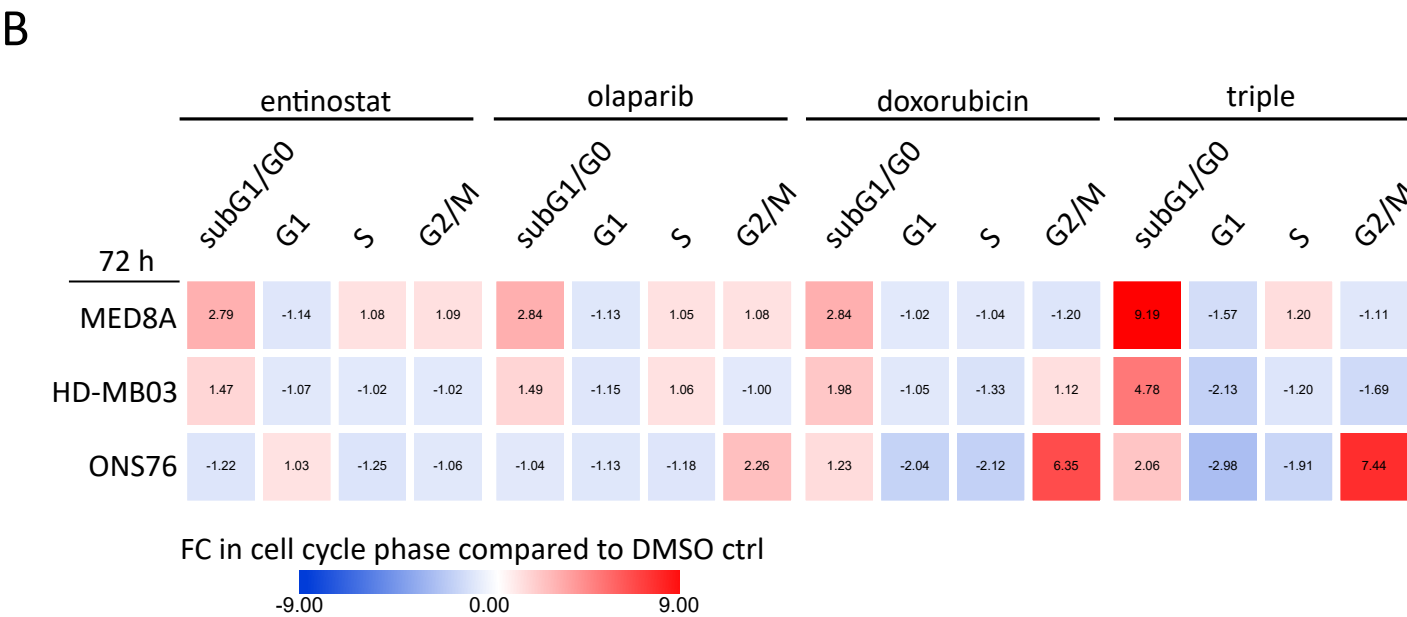

Supplement: Supplementary file 1 — Supplementary file1 (PDF 314 KB) Figure S1 EnrichmentMap for of GSEA of DEGOA. Results of the GSEA for KEGG, Reactome and GO-Database in DEGOA. Enriched gene sets with FDR < 0.1 are visualized through biofunctional clustering using EnrichmentMap. Positively enriched gene sets shown in red, negatively enriched gene sets in blue. Gene sets with overlap in genes cluster together. Dot size correlates with gene set size. Biomechanisms highlighted in yellow are targeted by the final five compounds. Figure S2. Single treatment dose response profiles of idasanutlin, olaparib, selinexor, ribociclib, vinblastine and the PARPi niraparib, pamiparib, veliparib in MB cell lines. A: Single dose-response curves of metabolic activity read-out after 72h treatment with idasanutlin, olaparib, ribociclib, selinexor, vinblastine in MYC-amplified (warm colors) and non-MYC-amplified MB cell lines (grey shades). Depicted are mean values ± SD relative to DMSO solvent control. Relative IC50 values of each drug in the corresponding cell line are listed in grey boxes. B: Comparison of IC50 values (dots, left y-axis) between MYC-amplified (left) and non-MYC-amplified MB cell lines (right) for idasanutlin, olaparib, selinexor, ribociclib and vinblastine. C: Single dose-response curves of metabolic activity read-out after 72h treatment with and niraparib, pamiparib, veliparib, legend and statistics as in (A) Figure S3 Quantification of DNA damage by γH2A.X staining. A: Bar graph depicts densitometric analysis of PARP1 signal intensity relative to actin in three biological replicates of western blot shown in Fig. 4B Significance calculated comparing each column to the 24h untreated condition, *p < 0.05, ns = not significant, (one way ANOVA). B: Box-dot plot comparing PARP2 mRNA expression in primary MB group3/4 subtypes. Dots represent single samples. Boxes extend from 25-75th percentiles, whiskers extend from 5-95th percentiles, lines at median. *p < 0.05, **p < 0.01; ***p < 0.001; ns not signific [file 11060_2023_4445_MOESM1_ESM.pdf]
